# Supplementary material for: The mutation rate of SARS-CoV-2 is highly variable between sites and is influenced by sequence context, genomic region, and RNA structure
Source: Nucleic Acids Res. 2025 Jun 12;53(11):gkaf503. doi: 10.1093/nar/gkaf503 (PMC12159741; doi:10.1093/nar/gkaf503)
Supplement: gkaf503_Supplemental_File [file gkaf503_supplemental_file.pdf]

Supplementary Information for: The mutation rate of SARS-CoV-2 is highly variable between sites and is influenced by sequence context, genomic region, and RNA structure

Hugh K. Haddox, Georg Angehrn, Luca Sesta, Chris Jennings-Shaffer, Seth D. Temple, Jared G. Galloway, Angie S. Hinrichs, William S. DeWitt, Jesse D. Bloom, Frederick A. Matsen IV, Richard A. Neher

April 28, 2025

Figures and Tables

mutation type	number of sites	remaining variance τ^2
A→C	1,479	0.519
A→G	2,426	0.227
A→T	1,291	0.669
C→A	649	0.488
C→G	532	0.886
C→T	1,539	0.476
G→A	862	0.350
G→C	261	1.167
G→T	261	0.971
T→A	2,280	0.783
T→C	4,430	0.276
T→G	2,032	0.618

Table S1: Characteristics and summary statistics of different mutation types. Column 1 lists the number of sites that met the criteria described in Figure 1A and are used in the estimation of the model. Column 2 shows the remaining variance in logarithmic count of mutations at these sites that is not explained by the model. tab:n sites

References

[1] Jesse D Bloom and Richard A Neher. Fitness effects of mutations to SARS-CoV-2 proteins. *Virus Evolution*, 9(2):vead055, 2023.

[2] Hagit T Porath, Shai Carmi, and Erez Y Levanon. A genome-wide map of hyper-edited RNA reveals numerous new sites. *Nature Communications*, 5(1):4726, 2014.

[3] Ernesto Picardi, Luigi Mansi, and Graziano Pesole. Detection of A-to-I RNA editing in SARS-CoV-2. *Genes*, 13(1):41, 2021.

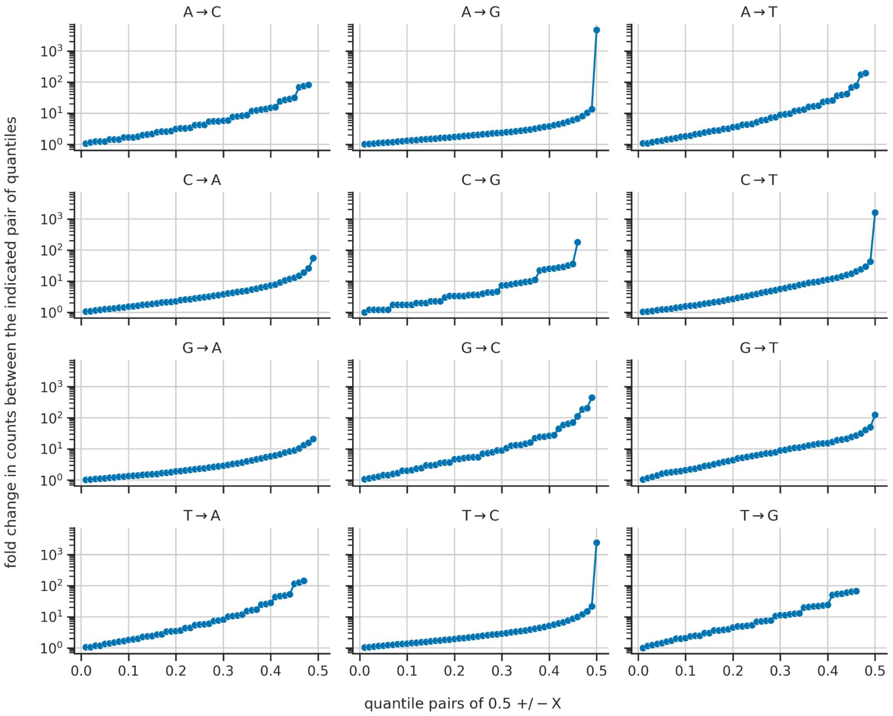

Figure S1: Fraction of sites with large fold differences in synonymous mutation counts. For each of the distributions plotted in Figure 1C, we systematically plot the fold change of mutation counts for quantile pairs that are equidistant from the median. We define pairs as 0.5 +/− X, where X ranges from 0.01 to 0.5 in steps of 0.01. For instance, X = 0.25 corresponds to the quantile pair 0.25 and 0.75, i.e., the interquartile range. For each pair, we then quantify the fold change in synonymous mutation counts per site between the upper and lower quantile in that pair. For some pairs, the lower quantile corresponds to zero counts, in which case we omit that pair from the plot. From the plot, it is possible to identify the lowest value of X that gives rise to a fold difference of interest (e.g., 10- or 100-fold) for a given mutation type. The fraction of sites that fall outside of this range are the fraction of sites with fold differences that are at least that extreme. For instance, for G→T mutations, there is a roughly 10-fold difference in counts between the 0.2 and 0.8 quantiles (X = 0.3). This indicates that 20% of sites on either side of the distribution differ by at least 10-fold. Similar evaluations can be made for any mutation type and any fold difference of interest.

fig:inter˙quantile˙ranges

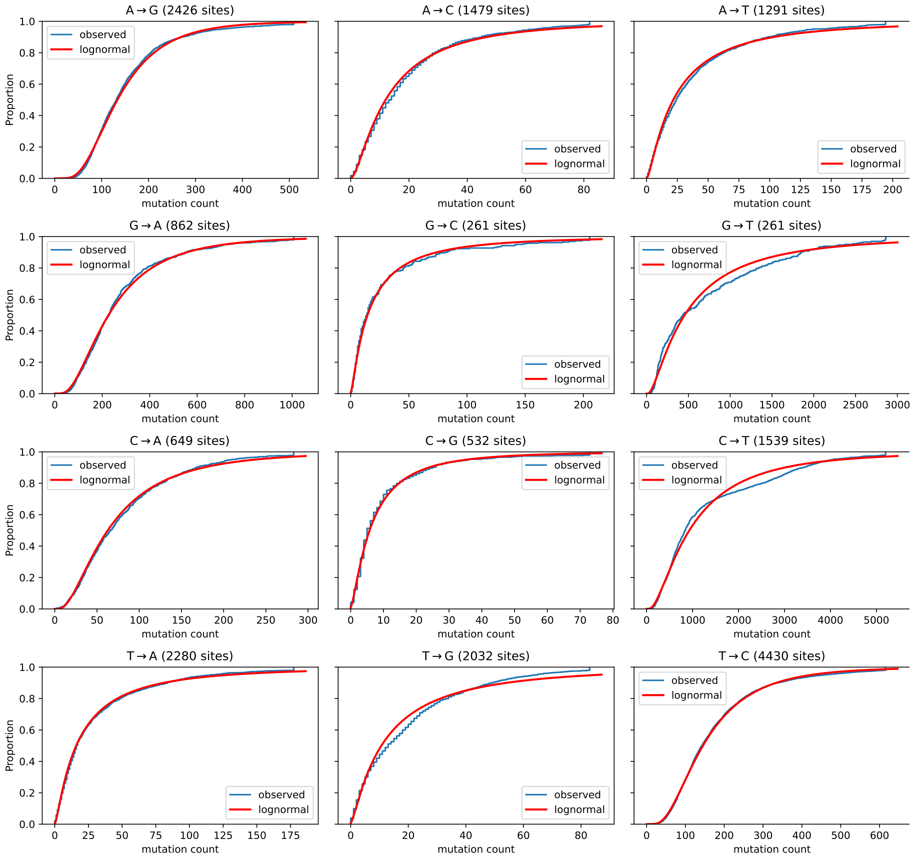

Figure S2: For each mutation type, the synonymous mutation counts per site closely follow a log-normal distribution. In each plot, the blue curve shows the cumulative distribution of the observed synonymous mutation counts per site for a given mutation type, while the red curve shows the cumulative distribution of a log-normal distribution with the same mean and variance as the corresponding observed distribution. In each case, the blue curve closely tracks with the red curve, with only a few deviations, showing that the observed counts closely follow a log-normal distribution.

fig:log˙normal

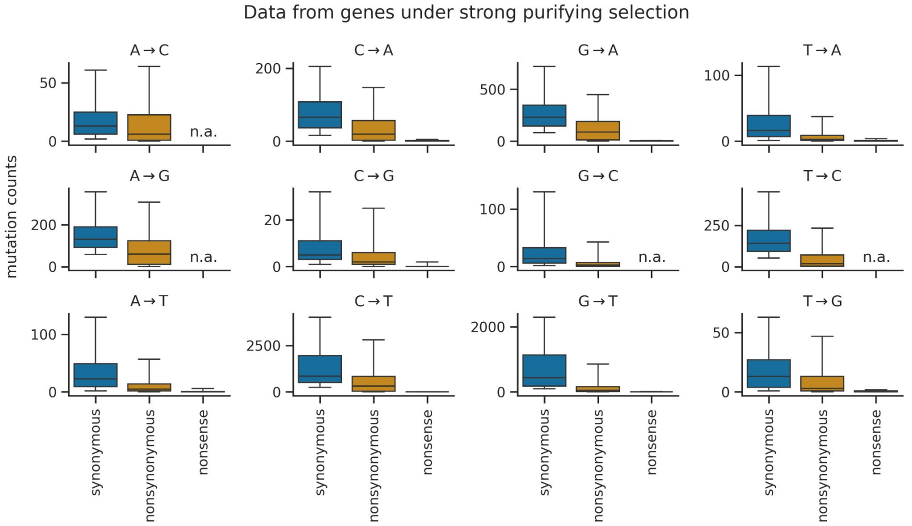

Figure S3: Distributions of site-specific mutation counts for synonymous, nonsynonymous, and nonsense mutations for each mutation type. To examine the possibility that artificial errors could impact the counts, we computed site-specific mutation counts for nonsynonymous and nonsense mutations using the same basic approach that we used for synonymous mutations. For each mutation type, box plots show site-specific mutation counts for synonymous, nonsynonymous, or nonsense mutations (whiskers show the 5th and 95th percentiles). Plots only show data for mutations in genes under strong purifying selection according to [1] (ORF1a, ORF1b, S, E, M, N), which make up most of the genome. Given the structure of the genetic code, some mutation types never lead to a stop codon in any context, and the plots for these mutation types have "n.a." instead of counts data for nonsense mutations. If the observed counts of *synonymous* mutations are inflated by artificial errors, then we would expect counts of nonsynonymous and nonsense mutations to be inflated by the same amount, as artificial errors are not subject to natural selection. On the other hand, if the observed mutation counts actually correspond to mutations that arose in globally circulating viruses and have been subjected to natural selection, we would expect the counts for nonsynonymous and nonsense mutations to be lower than counts for synonymous mutations, as nonsynonymous mutations are often deleterious and nonsense mutations are almost always highly deleterious. Overall, the distributions are consistent with the expected action of natural selection. Notably, nearly all nonsense mutations have counts near zero, indicating that artificial errors probably do not substantially inflate synonymous mutation counts in most cases. fig:mut˙counts˙supp

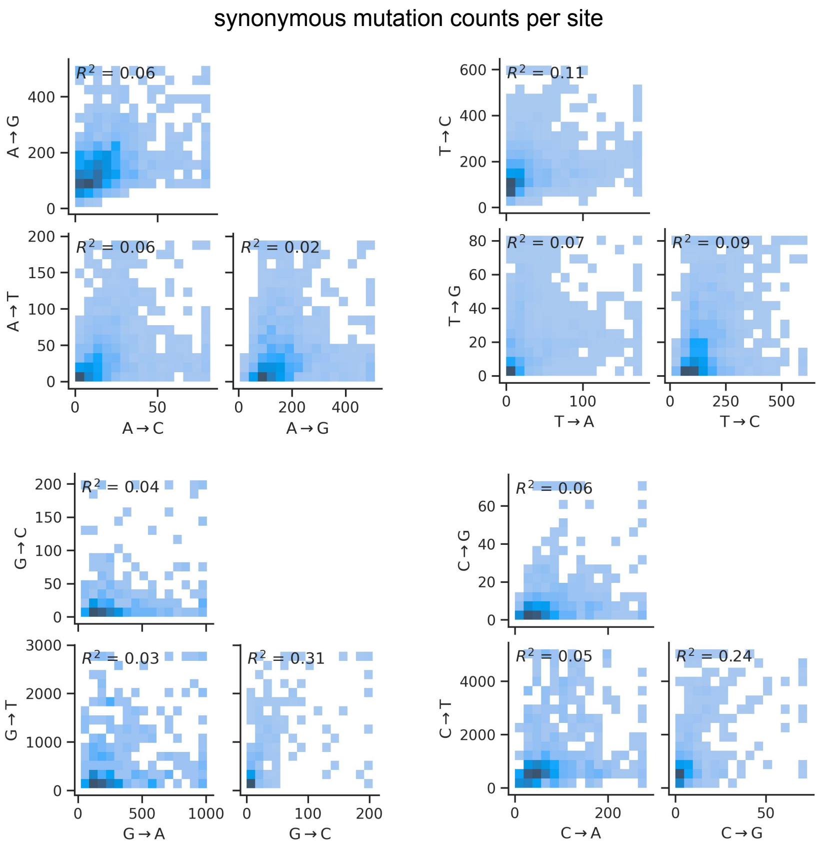

Figure S4: Correlation of synonymous mutation counts between different mutation types at the same site. For each mutation type, we apply a ceiling to the counts distribution at its 98th percentile. For most mutation types, the correlation is low, with $R^2 < 0.1$. Though, for a few pairs of mutation types (G→T and G→C or C→T and C→G), the correlation is substantially higher, indicating that counts of these pairs are more strongly influenced by common factors. One factor might be RNA structure, as these are the four mutation types where RNA structure has the largest effect on counts (Figure 2E).

fig:mut˙type˙corr

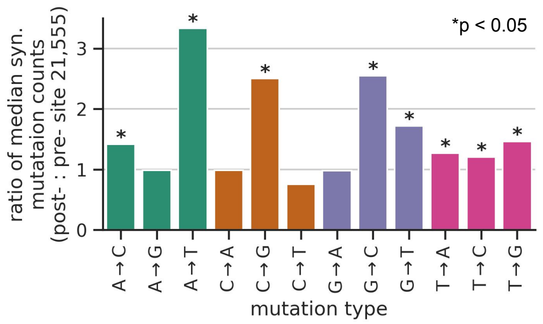

Figure S5: Position dependence of A→T mutation counts is also observed for C→G and G→C. The plot shows the ratio of median synonymous mutation counts between sites before vs. after site 21,555 in the genome. Asterisks indicate mutation types where the ratio of medians is significantly >1.0, as determined by randomization testing (see *Methods*), using a threshold of p<0.05 after Bonferroni correction for multiple-hypothesis testing. The largest effect sizes are for A→T, C→G and G→C. Some of the other mutation types show significant effects, but the effect sizes are not as large.

fig:genomic˙region˙pattern

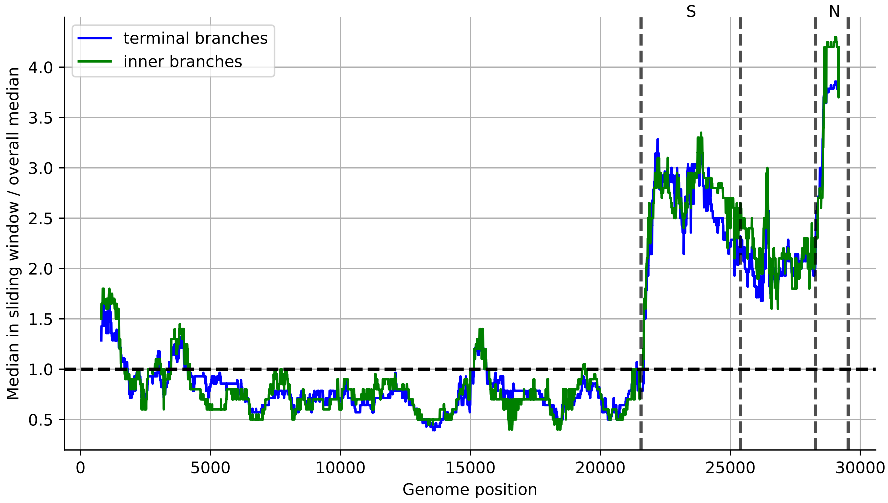

Figure S6: Position dependence of A→T mutation counts is observed for terminal and non-terminal mutations. The sharp increase of A→T mutation counts at the boundary between ORF1ab and S is not restricted to terminal mutations. Going from left to right, the first and second vertical lines mark the beginning and end of the S coding sequence, while the third and fourth vertical lines mark the beginning and end of the N coding sequence.

fig:AT˙terminal˙nonterminal

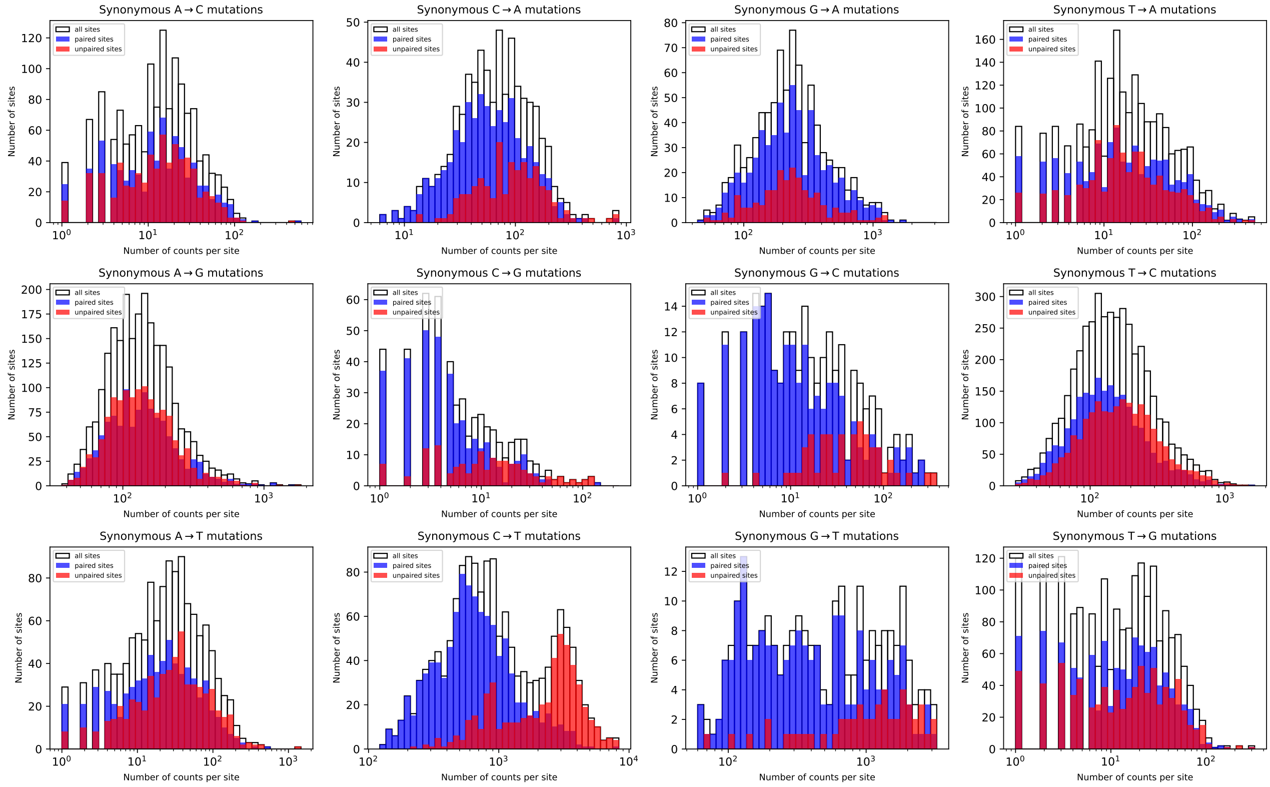

Figure S7: Distributions of mutation counts at sites that are base-paired or unpaired in the predicted secondary structure of the SARS-CoV-2 genome. Each panel shows the distribution of the mutation counts split by pairing state. Note that for some mutations, C→T in particular, the distribution of counts at unpaired sites is shifted to higher values compared to that unpaired sites.

fig:paired˙unpaired

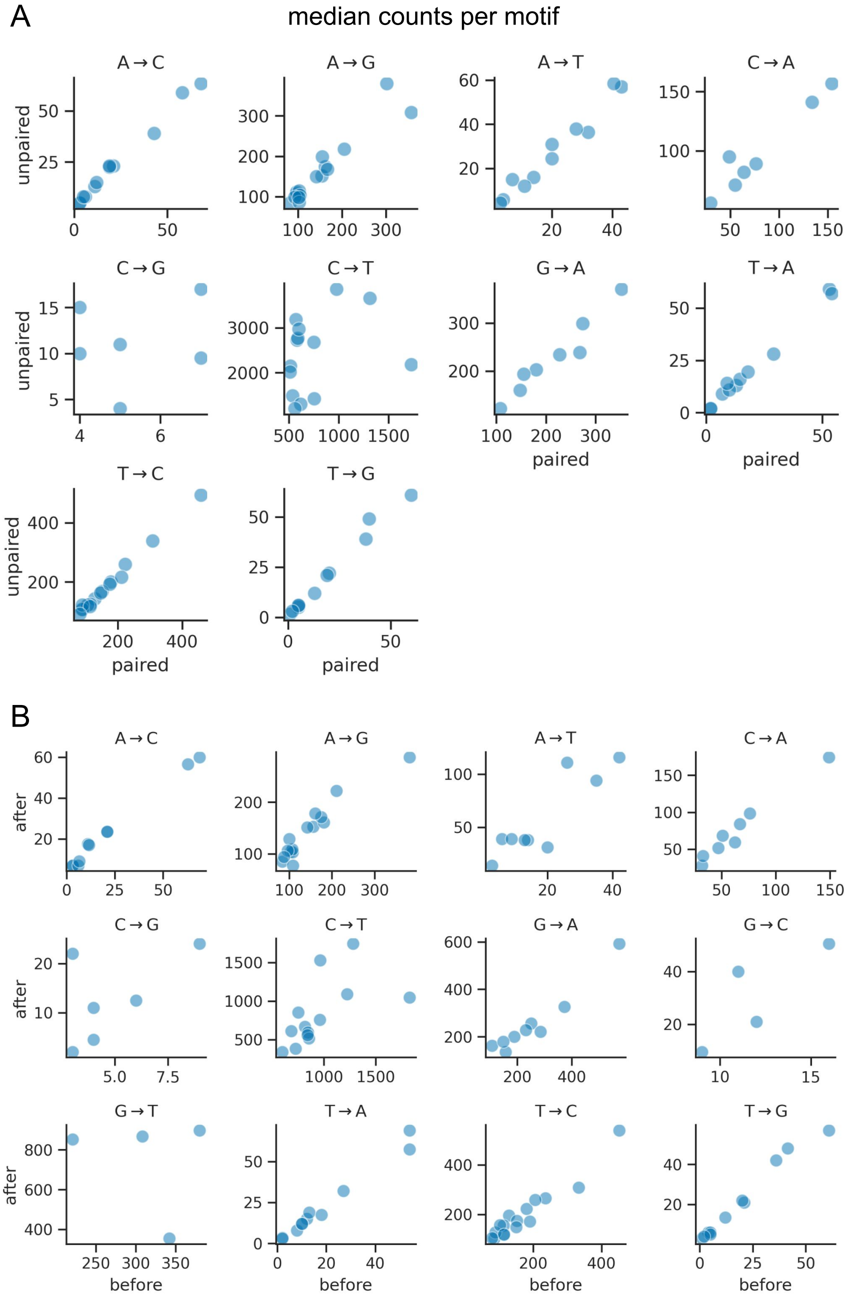

Figure S8: Correlation of 3-mer motif median synonymous mutation counts between subsets of sites in the genome for a given mutation type. (A) Comparing sites that are paired vs. unpaired in the predicted secondary structure. **(B)** Comparing sites before vs. after nucleotide 21,555 in the SARS-CoV-2 genome, which marks the end of ORF1b, the point at which synonymous mutation counts for A→T increase dramatically. These plots only show data for motifs with at least 10 sites in each subset for a given mutation type (a few mutation types do not have any motifs meeting this criterion). fig:local˙context˙differences

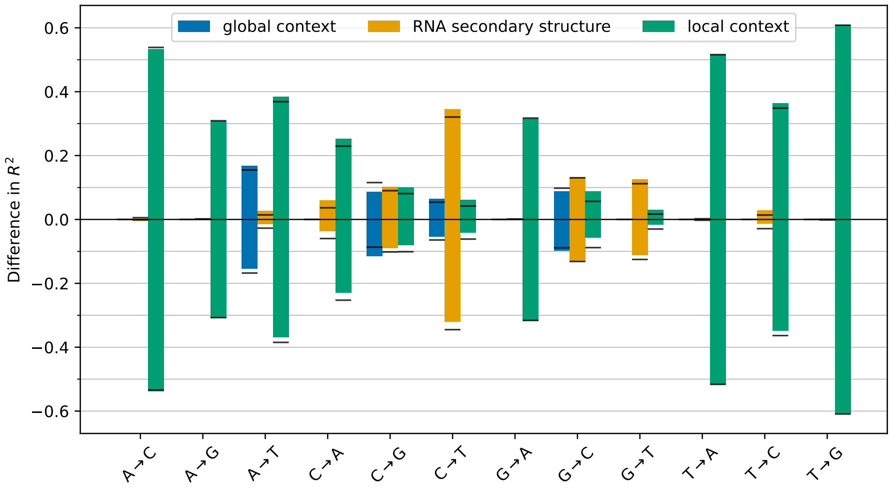

Figure S9: Individual contributions of different features are largely independent. The bars show the increments and decrements of the explained variance when features are used in isolation or removed from the full model, respectively. The horizontal black lines show the value of each bar mirrored across the x-axis. In almost all cases, the decrement upon removal matches their individual contribution, showing that these features are non-redundant. fig:separately˙R2

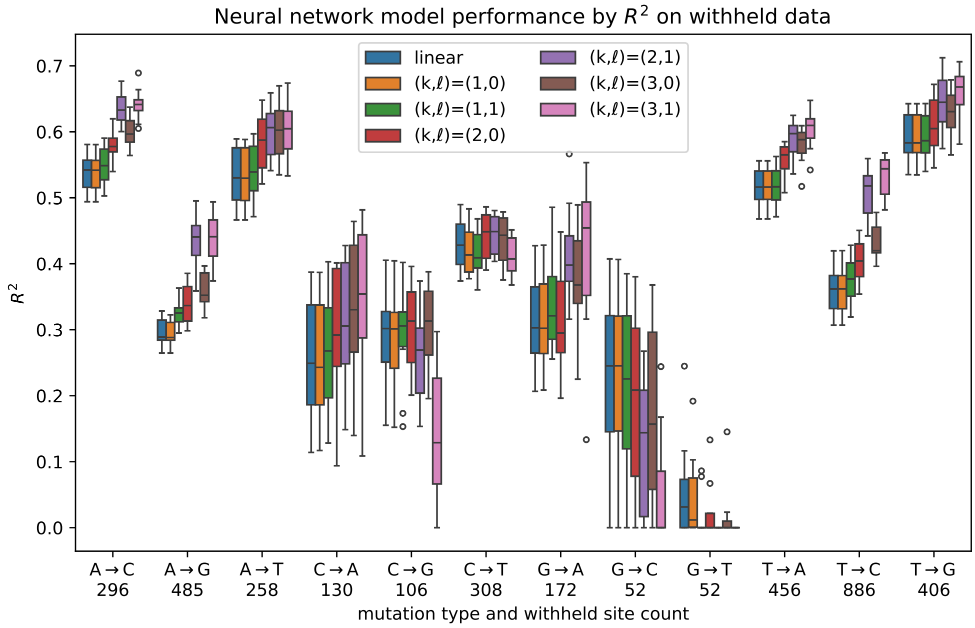

Figure S10: Performance of neural-network models and the linear model from the main text in their ability to predict site-specific synonymous mutation counts. We devised a neural-network architecture for predicting counts based on a site's genomic region, RNA structure, and local context (see *Methods*). We examined a series of models based on this architecture that integrate information over larger and larger windows of local sequence context (Fig. S11). The legend refers to these models using the convention described in the *Methods* and Fig. S11 (models with k of 1, 2 or 3 integrate information across sequence windows of length 3, 5, or 7, respectively). The "linear" model in the legend refers to the linear model from the main text. For each mutation type, we trained each model using a random 80% of sites and tested the model using the withheld 20% of sites, computing the R^2 between the predicted and observed counts from the withheld data. We did so using 10 randomly generated train-test splits, and each box plot shows the distribution of the resulting 10 R^2 values. For mutation types with more data (see the x-axis for the number of withheld sites per mutation type), the more complicated neural-network models (larger k and ℓ values) tended perform better than the linear model (higher R^2). However, for mutation types with less data, their performance was worse, suggesting more overfitting. Even when the neural networks performed better, the linear model still captured most of the variance captured by the neural networks, prompting us to use the linear model for estimating fitness effects.

fig:nn˙model˙r2

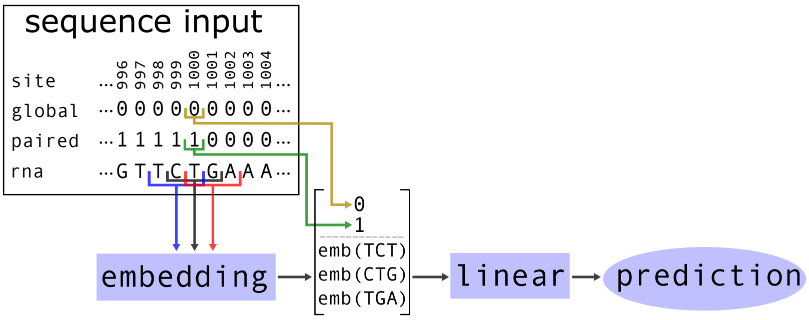

Figure S11: Summary of neural-network architecture. This figure shows an architecture where $k = 2$, $\ell = 1$ (see *Methods*), and how this architecture integrates information about local sequence context, RNA pairing state, and genomic (global) region, for site 1000. There are three layers: an embedding layer of length 3 motifs, a linear on the embedding and additional features, and lastly a prediction layer. In this example, the length 5 motif centered at site 1000 is represented by the length 3 motifs centered at sites 999, 1000, and 1001.

fig:nn˙model˙architecture

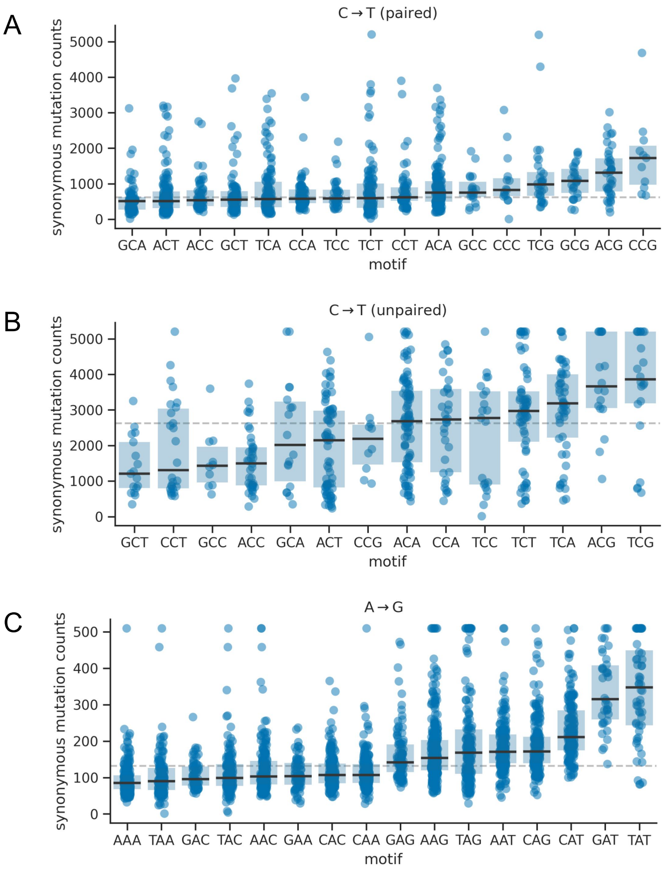

Figure S12: Effect of local sequence context on synonymous rates for additional mutation types. The panels in this figure are similar to the ones from Figure 3A/C. **(A)** and **(B)** separately show data for C→T mutations at sites that are paired or unpaired in the predicted secondary structure of the SARS-CoV-2 genome. For both sets of sites, there are only modest differences in the median mutation counts between different 3-mer motifs. **(C)** shows data for A→G mutations. A previous study identified putative ADAR1 motifs as being depleted in G nucleotides one base upstream of the mutated site and enriched in G nucleotides one base downstream of the site [2]. Another study identified the motifs as also being enriched in A nucleotides one base upstream and enriched in A nucleotides one base downstream [3]. These putative motifs do not clearly correspond to the ones with the highest counts. fig:local context ct ag

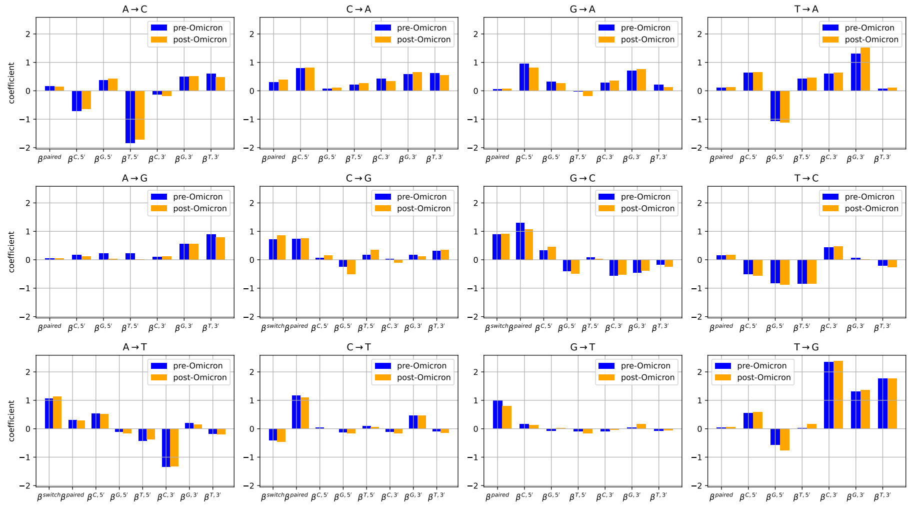

Figure S13: Comparison of model parameters inferred from data before and after the transition to Omicron variants. The effect of sequence context, RNA secondary structure, and large scale genomic regions is very similar for Omicron and pre-omicron variants.

fig:pre˙vs˙omicron
